# Protective and Antioxidant Effects of a Chalconoid from *Pulicaria incisa* on Brain Astrocytes

**DOI:** 10.1155/2013/694398

**Published:** 2013-08-28

**Authors:** Anat Elmann, Alona Telerman, Hilla Erlank, Sharon Mordechay, Miriam Rindner, Rivka Ofir, Yoel Kashman

**Affiliations:** ^1^Department of Food Quality and Safety, Volcani Center, Agricultural Research Organization, 50250 Bet Dagan, Israel; ^2^Dead Sea & Arava Science Center and The Shraga Segal Department of Microbiology & Immunology & Genetics, Ben-Gurion University of the Negev, 84105 Beer-Sheva, Israel; ^3^School of Chemistry, Tel Aviv University, 69978 Ramat Aviv, Israel

## Abstract

Oxidative stress is involved in the pathogenesis of neurodegenerative diseases such as Parkinson's and Alzheimer's diseases. Astrocytes, the most abundant glial cells in the brain, protect neurons from reactive oxygen species (ROS) and provide them with trophic support, such as glial-derived neurotrophic factor (GDNF). Thus, any damage to astrocytes will affect neuronal survival. In the present study, by activity-guided fractionation, we have purified from the desert plant *Pulicaria incisa* two protective compounds and determined their structures by spectroscopic methods. The compounds were found to be new chalcones—pulichalconoid B and pulichalconoid C. This is the first study to characterize the antioxidant and protective effects of these compounds in any biological system. Using primary cultures of astrocytes, we have found that pulichalconoid B attenuated the accumulation of ROS following treatment of these cells with hydrogen peroxide by 89% and prevented 89% of the H_2_O_2_-induced death of astrocytes. Pulichalconoid B exhibited an antioxidant effect both *in vitro* and in the cellular antioxidant assay in astrocytes and microglial cells. Pulichalconoid B also caused a fourfold increase in *GDNF transcription* in these cells. Thus, this chalcone deserves further studies in order to evaluate if beneficial therapeutic effect exists.

## 1. Introduction

Oxidative stress underlies the pathogenesis of a broad range of human diseases and in particular neurodegenerative disorders that are characterized by selective neuronal death [1–4]. The high vulnerability of the brain to oxidative damage is a contributing factor in the etiology of neurological and neuroinflammatory disorders. The brain is the most susceptible organ to oxidative damage because of its high oxygen demand and the high amounts of polyunsaturated fatty acids (PUFAs) present in neuronal membranes [5, 6]. In addition, certain regions of the brain are rich in iron, a metal that is catalytically involved in the production of damaging reactive oxygen species (ROS) [7]. Although ROS are critical intracellular signaling molecules [8], an excess of free radicals may lead to peroxidative impairment of membrane lipids and, consequently, to disruption of neuronal functions and apoptosis. The ROS known to be responsible for neurotoxicity are hydrogen peroxide (H_2_O_2_), superoxide anions (O_2_^−^), and hydroxyl radicals (OH). H_2_O_2_ is the major precursor of highly reactive free radicals that may cause damage in the cell, through iron ion- or copper ion-mediated oxidation of lipids, proteins, and nucleic acids. Excess of H_2_O_2_ accumulates during brain injuries and neurodegenerative diseases and can cross cell membranes to elicit harmful biological effects inside the cells [9]. This capability can partially account for H_2_O_2_-mediated neuronal [10] and astrocytic cell death [11]. Astrocytes play key roles in both normal and pathological brain functioning, making them viable targets for manipulation after brain injury. Astrocytes are responsible for the maintenance of brain homeostasis, and neurons are strictly dependent on astrocytes for their antioxidant redox status and survival [12]. Astrocytes also release multiple neurotrophic factors, such as GDNF, to protect themselves and neighboring cells [13]. Despite their high antioxidative activities, astrocytes exhibit a high degree of vulnerability and are not resistant to the effects of ROS. They respond to substantial or sustained oxidative stress with increased intracellular Ca^2+^, loss of mitochondrial potential, and decreased oxidative phosphorylation [14]. Since astrocytes form a tight functional unit with neurons and determine the brain's vulnerability to oxidative injury, impaired astrocytic energy metabolism and antioxidant capacity and the death of astrocytes may critically impair neuronal survival [15, 16]. Thus, protection of astrocytes from oxidative stress appears essential for the maintenance of brain functions.

Plants were shown to contain various bioactive compounds that might be developed as potential drugs. *Pulicaria incisa *(Lam.) DC. is a desert plant which belongs to the Asteraceae family and has been used for many years in traditional medicine for treatment of heart diseases and as a hypoglycemic agent [17–19]. An infusion prepared from *Pulicaria incisa *(*Pi*) is consumed instead of tea by many Egyptian Bedouins [18–20]. *Pi* has been found to contain high amounts of unsaturated fatty acids [21] to decrease total lipid, total cholesterol, and triglyceride levels and has been proposed as a potential hypocholesterolemic agent [22].

In a previous study, we have demonstrated that extracts prepared from *Pi* protected cultured astrocytes from oxidative stress-induced cell death [23]. The aim of this study was to isolate from *Pi* the bioactive compound that protect astrocytes from H_2_O_2_-induced cell death, to identify its structure and to characterize its antioxidant and protective activity in cultured primary astrocytes.

## 2. Materials and Methods

### 2.1. General Experimental Procedures

IR spectra were obtained with a Bruker FTIR Vector 22 spectrometer. ^1^H and ^13^C NMR spectra were recorded on Bruker Avance-500 spectrometer. COSY, HMQC, and HMBC experiments were recorded using standard Bruker pulse sequences. Electrospray MS (HRESIMS) measurements were performed on Waters Micromass SYNAPT HDMS Mass spectrometer (TOF). ^1^H and ^13^C NMR data are presented in Table 1.

Dulbecco's modified Eagle's medium (DMEM), RPMI-1640, Leibovitz-15 medium, glutamine, antibiotics (10,000 IU/mL penicillin and 10,000 *μ*g/mL streptomycin), soybean trypsin inhibitor, fetal bovine serum (FBS), and Dulbecco's phosphate buffered saline (PBS) (without calcium and magnesium) were purchased from Biological Industries (Beit Haemek, Israel); 2-mercaptoethanol, gelatin, crystal violet, Quercetin (3,3′,4′,5,7-pentahydroxyflavone), and 2′7′-dichlorofluorescein diacetate (DCF-DA) were purchased from Sigma Chemical Co. (St. Louis, MO, USA). 2,2′-Azobis (amidinopropane) (ABAP) was obtained from Wako Chemicals (Richmond, VA). Dimethyl sulphoxide (DMSO) was obtained from Applichem (Darmstadt, Germany); and hydrogen peroxide (H_2_O_2_) was obtained from MP Biomedicals (Ohio, USA). Crystal violet RNeasy Plus Mini Kit (Qiagen,  Hilden,  Germany), Thermo Scientific Verso cDNA kit (Thermo Fisher Scientific Inc), and TaqMan Gene Expression Assay from Applied Biosystems.

### 2.2. Plant Material

*Pulicaria incisa* were collected in the Arava Valley, and the voucher specimens have been kept and authenticated as part of the Arava Rift Valley Plant Collection (VPC) (Dead Sea and Arava Science Center, Central Arava Branch, Israel, http://www.deadseaarava-rd.co.il/) under the accession code AVPC0193.

### 2.3. Extraction and Isolation

Because of the complex mixture of compounds in the aqueous extract, we preferred stepwise extraction according to polarity. Namely, the wild sun dried plant *Pulicaria incisa* (56 g, aerial parts) was homogenized and extracted with ethyl acetate (EA), EA : MeOH (1 : 1) and MeOH. The organic extracts were evaporated to yield the crude extracts, while the most active one was the EA one (1.08 gr). As the EA : MeOH and MeOH extracts were not active, they were left out.

The EA crude extract was chromatographed several times on Sephadex LH-20 columns, eluting with hexane/MeOH/CH_2_Cl_2_ (2 : 1 : 1) to afford compound **1** (10.5 mg) and compound **2** (50 mg) as yellowish powders.

 Compound **1** HRESIMS [M+H]^+^ *m/z* 321.0968 (calc. 321.0974), C_16_H_16_O_7_; *α*_*D*_ (MeOH) −8.9; IR *ν*_max⁡_ (cm^−1^) 3577, 3492, 1626, 1460, and 1160; for NMR data see Table 1.
 Compound **2** HRESIMS [M+H]^+^ *m/z* 337.0918 (calc. 337.0923), C_16_H_16_O_8_; *α*_*D*_ (MeOH) −4.1; IR *ν*_max⁡_ (cm^−1^) 3577, 3492, 1626, 1460, and 1160; for NMR data see Table 1.

### 2.4. Preparation of Primary Cultures of Glial Cells

Primary cultures of glial cells were prepared from cerebral cortices of 1-2-day-old neonatal Wistar rats as described [24, 25]. The research was conducted in accordance with the internationally accepted principles for laboratory animal use and care, as found in the US guidelines and was approved by the Institutional Animal Care and Use Committee of The Volcani Center, Agricultural Research Organization.

### 2.5. Determination of Cell Viability

Astrocytes were re-plated at 24-well PDL-coated plastic plates at a density of 1 × 10^5^/well, in DMEM w/o phenol red containing 2% FBS, 2 mM glutamine, 100 U/mL penicillin, and 100 *μ*g/mL streptomycin. Cell viability was determined using a commercial colorimetric assay (Roche Applied Science, Germany) according to the manufacturer's instructions. This assay is based on the measurement of lactate dehydrogenase (LDH) activity released from the cytosol of damaged cells into the incubation medium.

### 2.6. Cellular Antioxidant Activity of Pulichalconoid B

Intracellular ROS production was detected using the nonfluorescent cell permeating compound, 2′7′-dichlorofluorescein diacetate (DCF-DA). DCF-DA is hydrolyzed by intracellular esterases and then oxidized by ROS to a fluorescent compound 2′-7′-DCF. Peroxyl radicals are generated by thermolysis of 2,2′-azobis (amidinopropane) (ABAP) at physiological temperature. ABAP decomposes at approximately 1.36 × 10^−6^ s^−1^ at 37°C, producing at most 1 × 10^12^ radicals/mL/s [26–28]. Microglial cells (130,000 cells/well) or astrocytes (300,000 cells/well) were plated in DMEM containing 2% FBS, 2 mM glutamine, 100 U/mL penicillin, and 100 *μ*g/mL streptomycin, onto 24 well plates and were incubated for 1 h with pulichalconoid B. Then, cells were preloaded with DCF-DA for 30 min, and washed twice with PBS, and ABAP (0.6 mM final concentration) was then added. The fluorescence, which indicates ROS levels, was measured in a plate reader with excitation at 485 nm and emission at 520 nm. Cell viability was determined by a modification of the crystal violet assay [29].

### 2.7. Determination of the Free-Radical Scavenging Activity in the DPPH Assay

Antioxidant activity was measured using the 2,2-diphenyl-1-picryhydrazyl (DPPH) radical scavenging assay. Different concentrations of pulichalconoid B were added to 1 mL of DPPH (3.9 mg/100 mL methanol) in test tubes wrapped in aluminum foil. Absorbance (*A*) was measured at 517 nm after 8 min incubation in the dark. The scavenging ability (%) of the samples was calculated as (*A*_control_ − *A*_sample_)/*A*_control_ × 100).

### 2.8. Treatment of Astrocytes

The original medium of the cells was aspirated off, and fresh medium was added to the cells. Dilutions of pulichalconoid B first in DMSO and then in the growth medium were made freshly from stock solution just prior to each experiment and were used immediately. The final concentration of DMSO in the medium was 0.2%. Dilutions of H_2_O_2_ in the growth medium were made freshly from 30% stock solution just prior to each experiment and were used immediately. Each treatment was performed in quadrplicates.

### 2.9. Induction of GDNF in Astrocytes

Astrocytes were replatedat 6-well PDL-coated plastic plates at a density of 2 × 10^6^ cells/well, in DMEM/F12 containing 5% FBS, 2 mM glutamine, 100 U/mL penicillin, and 100 *μ*g/mL streptomycin. Twenty four h after plating, the original medium of the cells was aspirated off, and fresh medium was added to the cells. Cells were then lysed and collected using RLT buffer (RLT trade name, Qiagen,  Hilden,  Germany) containing 1% *β*ME for RNA extraction.

### 2.10. Quantitative Real-Time PCR Analysis

RNA was extracted by the RNeasy Plus Mini Kit (Qiagen,  Hilden,  Germany) according to the manufacturer's instructions. Genomic DNA was removed from the RNA samples by using 50 units of RNase-free DNaseI at 37°C for 1 h. RNA (20 *μ*g) was converted to cDNA using the Thermo Scientific Verso cDNA kit (Thermo Fisher Scientific Inc.) following the manufacturer's protocol. The cDNA was used for quantitative real-time PCR amplification with TaqMan chemistry (Applied Biosystems) using rat GDNF predesigned TaqMan Gene Expression Assay from Applied Biosystems (Assay ID Rn00569510). Values were normalized relative to *β*-Actin (Assay ID Rn00667869). All results from three technical replicates were normalized to *β*-Actin and expressed as relative expression ratios calculated (relative quantity, RQ) using the comparative method and based on the data that were created by the ABI PRISM 7700 Sequence Detection System (using version 1.6 software).

### 2.11. Data Analysis

Statistical analyses were performed with one-way ANOVA followed by the Tukey-Kramer multiple comparison tests using Graph Pad InStat 3 for Windows (GraphPad Software, San Diego, CA, USA).

## 3. Results and Discussion

### 3.1. Purification Process

In our effort to discover new low molecular weight compounds that can protect brain cells from oxidative stress, fractions obtained from *Pi* were submitted to bioassay-guided fractionation by using an *in vitro* model in which H_2_O_2_ was used to mimic oxidative injury and to induce astrocytic cell death. 

Bioguided chromatography of the ethyl acetate extract of the aerial parts of the plant *Pulicaria incisa* afforded two active compounds (**1** and **2**). Compound **1**, pulichalconoid C, C_16_H_16_O_7,_ formula was reached by its HRESIMS and 1D NMR data. Its IR spectrum showed bands for hydroxyl, conjugated carbonyl, and a phenyl group. The proton NMR (Table 1) revealed six aromatic protons, that is, two doublets with a mutual coupling constant of 1.6 Hz and a four proton AA′BB′ system, possessing a 8.2 Hz coupling constant as well as two coupled methinoxy protons (4.50 (d, *J* = 11.9 Hz) and 4.95 (d, *J* = 11.9 Hz)) (all mutual coupling constants (Table 1) were confirmed by H-H COSY correlations). The latter two methinoxy protons together with a coupled C=O 196.1s and two carbon doublets at 72.2 and 82.8 ppm, methinoxy carbon atoms, indicated the presence of a CO–CH(OH)–CH(OH)–subunit. The latter moiety was further supported by proper CH correlations (H to C, HMBC correlations, summarized in Table 1). Another characteristic signal was a methoxyl three proton singlet at 3.50 ppm. The remaining twelve sp^2^ carbon atoms (Table 1) have constructed one parahydroxy and one 2-methoxy-4.6-dihydroxyphenyl system. The former phenol group was characterized by the two proton doublets at 6.85 and 7.28 (*J* = 8.2 Hz) and the latter aromatic ring by the 1.6 Hz metacoupling. Both rings and their assembly in the molecule were fully supported by two and three bond CH correlations (H to C HMBC correlations, Table 1). As the methoxy carrying ring is not symmetric, the methoxy group has to be on the orthoposition. Next, based on the 11.9 Hz coupling constant between H-7 and H-8 a threoconfiguration is inferred to these two stereocenters of the molecule. Only antirelationship between H-7 and H-8, required for the anticonformers, agrees with the large coupling constant [30]. All the above information suggests for **1** the 7-(4-hydroxyphenyl)-7,8-dihydroxy-9-(2′,4′,-dihydroxy-6′-methoxyphenyl) propan-9-one structure.

**Figure 1:**
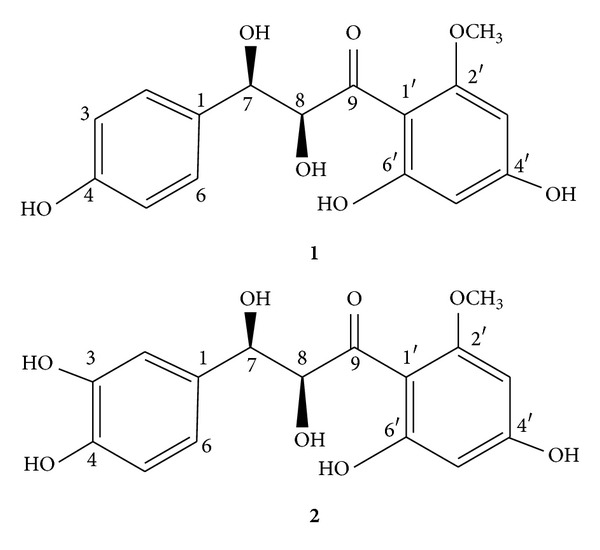
Structures of the active pulichalconoides B and C.

The second isolated compound (**2**), pulichalconoid B, C_16_H_16_O_8_ (HRESIMS), carrying an additional oxygen atom was found to be closely related to compound **1**. The difference between the two compounds being the replacement of the 4-hydroxyphenyl by a 3,4-dihydroxypheny group. As with **1**, the 1,3,4-substitution pattern of the latter ring was established by 1D and 2D NMR data (Table 1), namely, by the ortho- (8.0 Hz) and meta- (1.6 Hz) coupling constants as well as COSY and HMBC experiments (Table 1). The pure molecules have similar structure and differ only in one hydroxyl group (Figure 1).

Compounds **1** and **2** belong to the well-known flavanoids' precursors—the chalconoids. Chalcones are the natural precursors of flavonoids and isoflavonoids in higher plants [21, 31–33]. Concerning the chemical structural level, in these phenolic compounds, two aromatic rings are joined by a three-carbon *α*,*β*-dihydroxy carbonyl system [34–36]. Chalcones are unique in the flavonoids family in lacking a heterocyclic C ring. Although their importance to human health has remained unclear, several studies have shown that chalcones display a wide variety of biological and pharmacological proprieties that include antioxidant [37–39], anti-inflammatory [39–41], antiproliferative [37, 42], antiangiogenic [32, 42] and anticancer activities [36, 39, 43]. Chalcones are able to cross the blood brain barrier and have been proposed to play a useful role in protecting the central nervous system against oxidative and excitotoxic stress [38, 44–50]. Clinical trials have shown that these compounds reach reasonable plasma concentrations and are not associated to marked toxicity [51]. In this research, we have concentrated on studying the effects of pulichalconoid B because of its higher yield (16.7 mg versus 3.5 mg of compound pulichalconoid C).

### 3.2. Pulichalconoid B Protected Astrocytes against H_2_O_2_-Induced Cell Death

Astrocytes support the survival of neurons in the central nervous system, and any damage to them caused by oxidative stress would lead to neuronal death. In light of the fact that oxidative stress has become accepted as a target of therapeutic interventions for the treatment of brain injuries and neurodegenerative diseases and the critical role of astrocytes in neuronal survival, the present study examined the effects of pulichalconoid B on the susceptibility of astrocytes to oxidative stress. 

**Figure 2:**
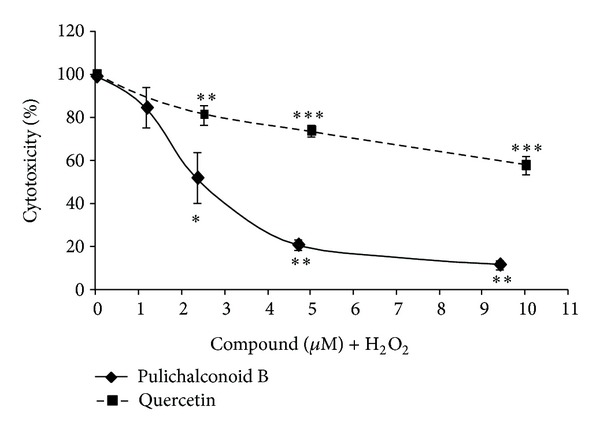
Pulichalconoid B protects astrocytes from H_2_O_2_-induced cell death. Astrocytes were treated with different concentrations of pulichalconoid B or quercetin. H_2_O_2_ (200 *μ*M) was added 2 h after the addition of pulichalconoid B or quercetin, and cell death was determined 20 h later. The results are means ± SEM of two experiments (*n* = 8). Statistical analyses were performed with one-way ANOVA followed by the Tukey-Kramer multiple comparison tests. **P < 0.01, ***P < 0.001.

H_2_O_2_ exposure is used as a model of ischemia reperfusion. The concentration of H_2_O_2_ used in our experiments (175–200 *μ*M) resembles the concentration reported in rat striatum under ischemic conditions [52]. In order to characterize the ability of pulichalconoid B to protect against H_2_O_2_-induced oxidative stress, we assessed changes in cell viability using a model in which oxidative stress was induced by the addition of H_2_O_2_ to cultures of primary astrocytes. Exposure of normal primary astrocytes to H_2_O_2_ resulted in a time- and concentration-dependent death of astrocytes at 20 h after exposure [53]. To determine the optimal concentration of pulichalconoid B needed for a protective effect, astrocytes were preincubated with different concentrations of this molecule. H_2_O_2_ was then added, and cytotoxicity was determined after 20 h. Our results show that pulichalconoid B exhibited a protective effect against H_2_O_2_-induced cell death, and was fully effective (89% protection) at 9 *μ*M (Figure 2). As a positive control, we have used the flavonoid quercetin. This flavonoid was studied in primary astrocytes and was found to be nontoxic up to 100 *μ*M [54]. At the same concentration (9 *μ*M), quercetin was less effective than pulichalconoid B and provided only 42% protection (Figure 2). 

**Figure 3:**
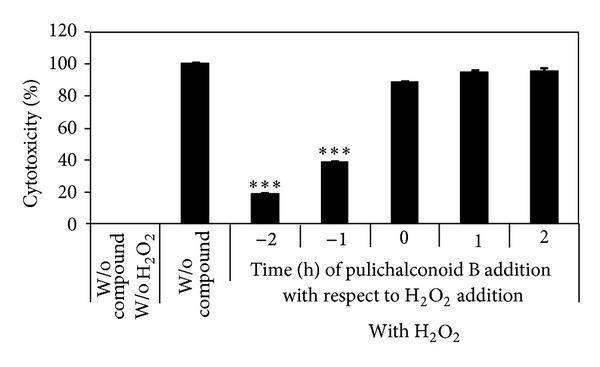
Preincubation of astrocytes with pulichalconoid B is a prerequisite for the protective effect against H_2_O_2_ cytotoxicity. Pulichalconoid B (6.25 *μ*M) was added to astrocytes before (−2 h, −1 h) concomitant (0) or after (+1 h, +2 h) the addition of H_2_O_2_. Cytotoxicity was measured 20 h later by the levels of LDH in the conditioned media. “W/o” means without. The results are means ± SEM of three experiments (*n* = 12). Statistical analyses were performed with one-way ANOVA followed by the Tukey-Kramer multiple comparison tests ***P < 0.001.

To determine the optimal timing of the application of the pulichalconoid B to attenuate the effect of H_2_O_2_, the cells were preincubated with pulichalconoid B for 1-2 h, co-treated with H_2_O_2_, or posttreated with H_2_O_2_. Our results demonstrate that pulichalconoid B is more effective when applied to the cells before H_2_O_2_ and that the addition of pulichalconoid B to the cells after the application of H_2_O_2_ did not reverse the toxic effect (Figure 3). This may indicate that pulichalconoid B sets the cells into a defensive state that helps them to contend with oxidative stress and that it takes about 1 h to get the cells into that defensive state. If this is the case, it indicates that pulichalconoid B is not only an anti-oxidant but also might bind to a cell surface or intracellular receptors and might enhance the resistance of astrocytes to H_2_O_2_ toxicity by affecting different processes induced by H_2_O_2_ [55–57].

### 3.3. Pulichalconoid B Inhibited the H_2_O_2_-Induced Generation of ROS

**Figure 4:**
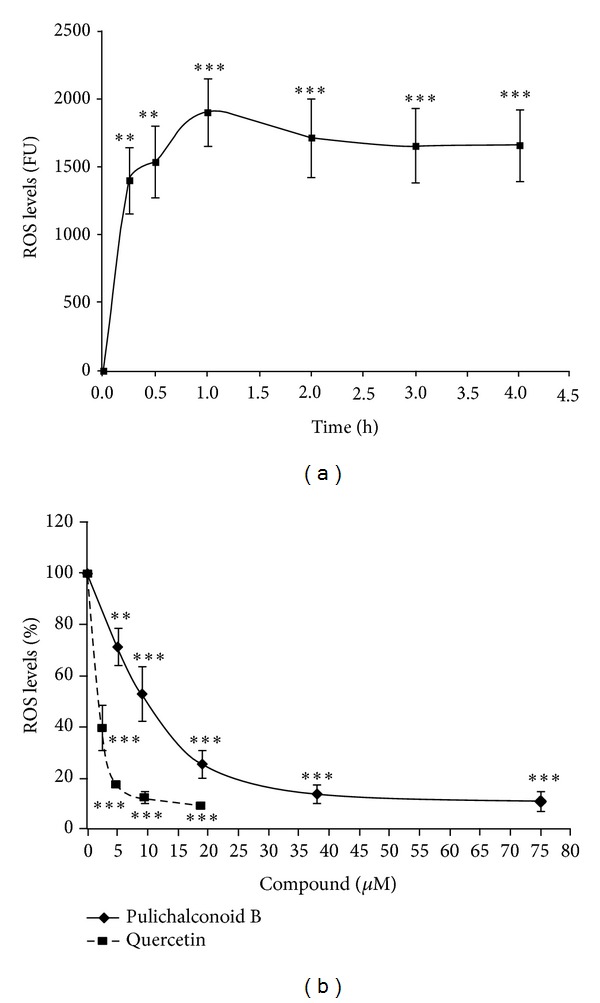
Pulichalconoid B attenuates H_2_O_2_-induced ROS production in astrocytes. Astrocytes were preloaded with DCF-DA for 30 min and washed. Preloaded astrocytes were then preincubated with pulichalconoid B or quercetin for 2 h. H_2_O_2_ was added to the culture and the fluorescence intensity representing ROS levels were measured. (a) The fluorescence levels (Fluorescence units, FU) of cells that had been treated with 175 *μ*M H_2_O_2_. ROS levels were measured at the indicated time points. (b) The ROS levels of cells that had been preincubated with various concentrations of pulichalconoid B or quercetin before H_2_O_2_ addition were measured after 1 h. The results represent the means ± SEM of two experiments (*n* = 7). Statistical analyses were performed with one-way ANOVA followed by the Tukey-Kramer multiple comparison tests **P < 0.01, ***P < 0.001.

H_2_O_2_-induced cell death is accompanied by an increase in ROS levels. In order to determine whether pulichalconoid B could inhibit the production of ROS that is induced by H_2_O_2_, we assessed the intracellular generation of ROS and tested whether treatment of astrocytes with pulichalconoid B affected their levels. For that purpose, the cells were preloaded with the ROS indicator DCF-DA and were treated with various concentrations of pulichalconoid B before the application of H_2_O_2_. ROS formation was determined by examining fluorescence every hour for 4 h. As shown in Figure 4(a), H_2_O_2_ induced production of ROS in astrocytes, with the maximum levels of ROS produced after 1 h. Pretreatment of astrocytes with pulichalconoid B inhibited the H_2_O_2_-induced elevation of the levels of intracellular ROS, with maximal inhibition of 89% after 1 h (Figure 4(b)). At the same experimental conditions, the lowest dose of quercetin that was tested in this study (2.4 *μ*M) was more effective than pulichalconoid B (*P* < 0.001) and provided 82% inhibition, while pulichalconoid B provided 29% inhibition at this concentration. However, there was no significant difference (*P* > 0.05) between the efficiencies of quercetin and pulichalconoid B (~88% inhibition) at the maximal doses that were tested in this study (Figure 4(b)). 

**Figure 5:**
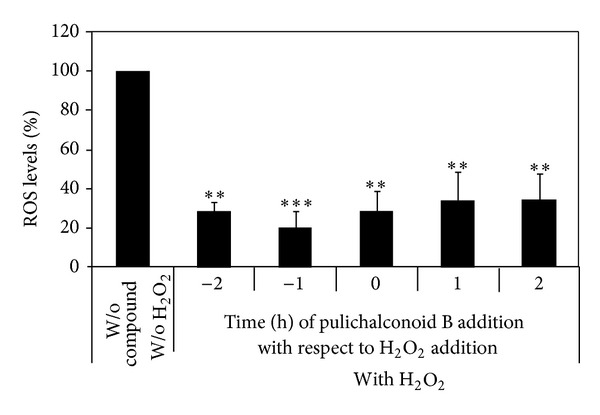
Pulichalconoid B is similarly effective in attenuating ROS production when applied before or after H_2_O_2_. Astrocytes were preloaded with DCF-DA for 30 min and washed. Pulichalconoid B (19 *μ*M) was added to astrocytes before (−2 h, −1 h) concomitant (0) or after (1 h, 2 h) the addition of H_2_O_2_ (175 *μ*M). ROS levels were measured 4 h after the application of H_2_O_2_. “W/o” means without. The results represent the means ± SEM of two experiments (*n* = 7). **P < 0.01, ***P < 0.001. Statistical analyses were performed with one-way ANOVA followed by the Tukey-Kramer multiple comparison tests.

To determine the time at which pulichalconoid B best ameliorates H_2_O_2_-induced ROS induction, the cells were pre-incubated with pulichalconoid B for 2 h or 1 h, cotreated with H_2_O_2_ and pulichalconoid B, or posttreated (for 2 h or 1 h) with pulichalconoid B. Interestingly, in contrast to the results obtained in our cell protection assay (Figure 3), pulichalconoid B was similarly effective in attenuating ROS production when it was preincubated with the cells and when it was applied after the H_2_O_2_ (Figure 5).

### 3.4. Pulichalconoid B Reduced 2,2′-Azobis- (Amidinopropane) (ABAP-) Mediated Peroxyl Radicals Levels in Astrocytes and Microglial Cells

**Figure 6:**
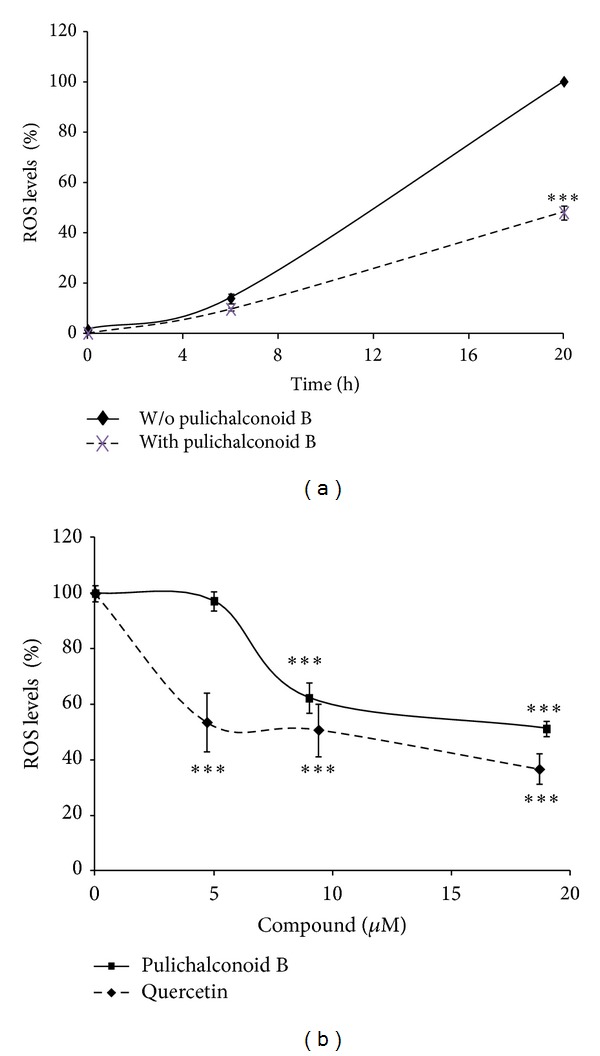
Pulichalconoid B inhibits peroxyl radical-induced oxidation of DCFH to DCF in primary astrocytes. Astrocytes were incubated with pulichalconoid B. Then, the astrocytes were preloaded with DCF-DA for 30 min and washed. ABAP (0.6 mM) was added to the culture, and the fluorescence intensity (representing ROS levels) was measured. (a) The fluorescence levels of cells that had been pre-incubated with 37.5 *μ*M pulichalconoid B were measured at the indicated time points. The results are the means ± SEM of three experiments (*n* = 12). (b) The fluorescence levels of cells that have been pre-incubated with various concentrations of pulichalconoid B were measured after 20 h. The results are the mean ± SEM of three experiments (*n* = 10). “W/o” means without. Statistical analyses were performed with one-way ANOVA followed by the Tukey-Kramer multiple comparison tests. ***P < 0.001.

**Figure 7:**
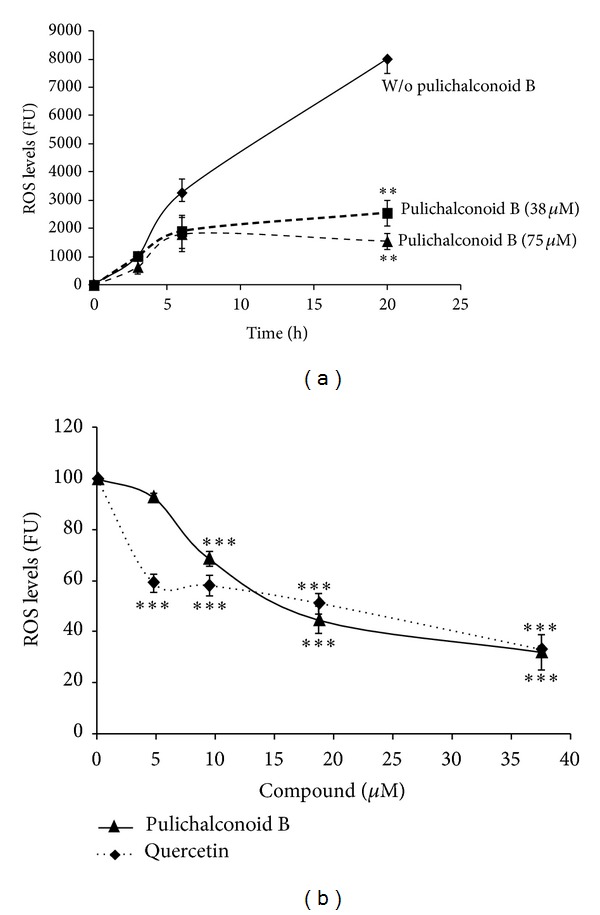
Pulichalconoid B inhibits peroxyl radical-induced oxidation of DCFH to DCF in primary microglial cells. Microglial cells were incubated for 1 h with pulichalconoid B or quercetin. Then, cells were preloaded with DCF-DA for 30 min and washed. ABAP (0.6 mM) was added to the culture, and the fluorescence intensity, representing ROS levels was measured. (a) The fluorescence levels (Fluorescence units, FU) of cells that had been preincubated with the indicated concentrations of pulichalconoid B were measured at the indicated time points. (b) ROS levels of cells that had been pre-incubated with various concentrations of pulichalconoid B or quercetin were measured after 20 h. The results represent the means ± SEM of two different experiments (*n* = 8). “W/o” means without. Statistical analyses were performed with one-way ANOVA followed by the Tukey-Kramer multiple comparison tests. ***P < 0.001.

In our model, there are two opportunities for pulichalconoid B to elicit antioxidant effects: it can act at the cell membrane to disrupt peroxyl radical chain reactions at the cell surface; or it can penetrate the cell and react with ROS inside the cell. In order to discriminate between these possibilities, we used a cellular antioxidant activity assay which measures the ability of pulichalconoid B to enter the cells and prevent the formation of DCF by ABAP-generated peroxyl radicals [58]. Astrocytes or microglial cells were preincubated with ABAP, which generates peroxyl radicals inside cells. The kinetics of DCFH oxidation in astrocytes and microglial cells by ROS generated from ABAP is shown in Figures 6(a) and 7(a), respectively. The increase in ROS-induced fluorescence was moderated by pulichalconoid B, as shown in Figure 6(b) for astrocytes (49% inhibition at the highest dose) and in Figure 7(b) for microglial cells (80% inhibition at the highest dose). At the lowest dose (5 *μ*M) tested, quercetin, which was used as a control compound, was more efficient than pulichalconoid B in reducing intracellular ROS levels in microglial cells (*P* < 0.001; Figure 7(b)) as well as in astrocytes (*P* < 0.001; Figure 6(b)). However, at higher doses, these compounds were similarly effective (*P* > 0.05). In this assay, the efficiency of cellular uptake and/or membrane binding combined with that of the radical-scavenging activity dictate the efficacy of the tested compound. The fact that pulichalconoid B inhibited intracellular ROS levels suggests that, in addition to other possible activities, this compound may enter glial cells, and maybe also the blood brain barrier (BBB), and react with ROS inside those cells.

### 3.5. Free-Radical Scavenging Activity of Pulichalconoid B

**Figure 8:**
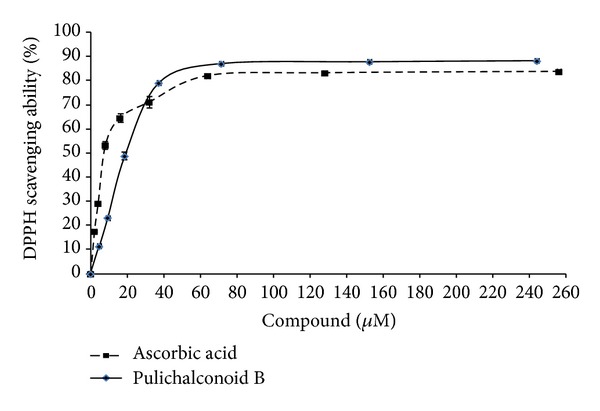
DPPH radical scavenging activity of pulichalconoid B. The DPPH scavenging ability of pulichalconoid B and ascorbic acid was compared for eight minutes. The results are presented as means ± SD (*n* = 2).

The antioxidant activity of pulichalconoid B was also demonstrated *in vitro*. The free-radical scavenging activity of pulichalconoid B was determined by the 2,2-diphenyl-1-picrylhydrazyl (DPPH) radical which is considered to be a model lipophilic radical. In this assay, pulichalconoid B was found to be a free-radical scavenger with an IC_50_ value of 17 *μ*M and 88% scavenging ability of DPPH (Figure 8).

### 3.6. Pulichalconoid B Stimulated GDNF Expression in Astrocytes

The contrast between the need for a pre-incubation of the cells with pulichalconoid B in order to gain the protective effect (Figure 3) and the fact that the timing of the addition of pulichalconoid B did not appear to affect the ability of this compound to neutralizing ROS levels (Figure 5) led us to examine whether another factor aside from antioxidant activity might be involved in the protective effects of pulichalconoid B. 

**Figure 9:**
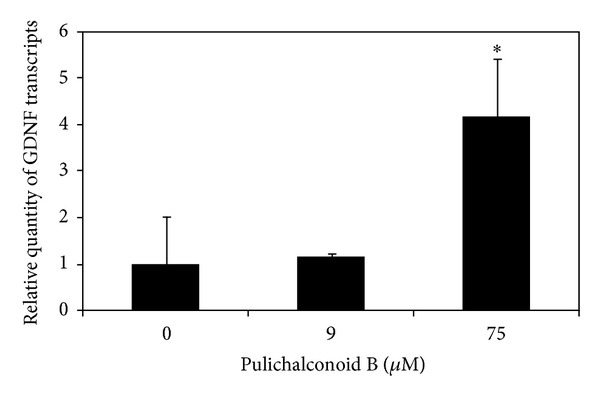
Treatment with pulichalconoid B increases the levels of *GDNF* transcript in primary astrocytes. Astrocytes were exposed to the indicated concentrations of pulichalconoid B for 5 h. *GDNF* transcripts were measured using quantitative real-time PCR. The results of three technical replicates were normalized to *β*-Actin and are expressed as relative quantities of *GDNF* transcripts. The results are means ± SD of one out of three experiments. The *t*-test was used to evaluate the effects of pulichalconoid B on the expression of *GDNF*. *P < 0.05.

It has been previously shown that GDNF protects astrocytes from ischemia-induced apoptosis [13]. Thus, we raised the possibility that the protective effect of pulichalconoid B on astrocytes suffering oxidative stress might be at least partially due to the induction of GDNF by pulichalconoid B. To test this possibility, primary astrocytes were treated with different concentrations of pulichalconoid B for several incubation periods, and then levels of *GDNF* transcripts were determined by real-time PCR and quantified. The results of these experiments showed that incubation with pulichalconoid B at an optimal concentration of 75 *μ*M leads to a 4-fold increase in *GDNF* mRNA levels following 5 h of incubation (Figure 9). The importance of these findings is emphasized by the fact that astrocyte-derived GDNF is a potent inhibitor of microglial activation [59]. Moreover, the neurotrophic and protective effects of *GDNF* on neuronal cells have made GDNF a promising candidate for gene and cell therapy for various neurodegenerative diseases [60–66].

## 4. Conclusions

Substances that can protect brain cells from oxidative stress are potential tools for the treatment of brain injuries and neurodegenerative diseases. In the present study, we evaluated the effectiveness of a new chalcone that we isolated from the desert plant *Pulicaria incisa*—pulichalconoid B—for counteracting oxidative damage in cultured glial cells. Pulichalconoid B protected astrocytes from H_2_O_2_-induced cell death and reduced the levels of intracellular ROS produced by astrocytes and microglial cells following treatment with H_2_O_2_ or ABAP. Pulichalconoid B is not a mere anti-oxidant and its beneficial effects are also demonstrated by the induction of GDNF expression by astrocytes. 

On the basis of the current results, we suggest that this new chalcone, which is characterized by low polarity and low molecular weight (320) might traverse the blood brain barrier (BBB) and affect different brain functions as was found for other chalcones. We suggest that pulichalconoid B should be further evaluated for the development of a drug for the prevention or treatment of brain injuries and neurodegenerative diseases which involve oxidative stress and astrocytic cell death. 

## Figures and Tables

**Table 1:** NMR data for compounds **1** and 2^*a*^.

Position	**1(C)**		**2(B)**		H to C
1	127.2 (qC)		127.8 (qC)		Correlations
2	129.0 (CH)	7.28 d (8.2)	114.4 (CH)	6.92 d (1.6)	4, 6, 7
3	115.0 (CH)	6.85 d (8.2)	144.5 (qC))		
4	157.3 (qC)		145.4 (qC)		
5	115.0 (CH)	6.85 d (8.2)	115.1 (CH)	6.80 d (8.0)	1, 3, 4
6	129.0 (CH)	7.28 d (8.2)	119.7 (CH)	6.82 dd (8.0, 1.6)	4, 7, 8
7	72.2 (CH)	4.50 d (11.9)	72.1 (CH)	4.44 d (12.4)	9, 8
8	82.8 (CH)	4.95 d (11.9)	83.4 (CH)	4.95 d (12.4)	7, 9
9	196.1 (qC)		196.5 (qC)		
1′	101.0 (qC)		101.0 (qC)		
2′	163.3 (qC)		163.3 (qC)		
3′	94.2 (CH)	5.98 d (1.6)	94.2 (CH)	5.92 d (1.8)	4′, 1′
4′	168.5 (qC)		168.3 (qC)		
5′	95.0 (CH)	6.05 d (1.6)	95.1 (CH)	5.95 d (1.8)	4′, 1′
6′	163.0 (qC)		162.7 (qC)		
OMe	55.0 (CH_3_)	3.50 s	55.5 (CH_3_)	3.75 s	3′, 4′

^a^Recorded at 125 MHz and 500 MHz for ^13^C and ^1^H, respectively, in CDCl_3_; *J *values in Hz in parenthesis.

## Abbreviations

ABAP: 2,2′-Azobis (amidinopropane)
DCF-DA: 2′7′-Dichlorofluorescein diacetate
DPPH: 2,2-Diphenyl-1-picryhydrazyl
FU: Fluorescence units
GDNF: Glial-derived neurotrophic factor
*Pi*: *Pulicaria incisa*
ROS: Reactive oxygen species
W/o: Without.
